# Association of anti SITH 1 antibody titer with mental stress and intracranial aneurysms

**DOI:** 10.1038/s41598-026-42027-8

**Published:** 2026-03-24

**Authors:** Michiyasu Fuga, Hirokazu Koseki, Nobuyuki Kobayashi, Toshihiro Ishibashi, Azusa Ishii, Naomi Oka, Tohru Sano, Naoki Kato, Ken Aoki, Shota Sonoda, Kyoko Ito, Toshihide Tanaka, Yuzuru Hasegawa, Shogo Kaku, Minoru Kogiku, Kazuhiro Kondo, Yuichi Murayama

**Affiliations:** 1 Department of Neurosurgery, The Jikei University School of Medicine, 3-25-8 Nishi-Shimbashi, Minato-ku, Tokyo, 105-8461 Japan; 2 Department of Neurosurgery, The Jikei University School of Medicine, Kashiwa Hospital, 163-1 Kashiwashita, Kashiwa-City, Chiba 277-8567 Japan; 3 Center for Preventive Medicine, The Jikei University Hospital, 3-19-18 Nishi- Shimbashi, Minato-City, Tokyo 105-8471 Japan; 4 Department of Virology, The Jikei University School of Medicine, 3-25-8 Nishi- Shimbashi, Minato-ku, Tokyo, 105-8461 Japan; 5 Department of Neurosurgery, The Jikei University School of Medicine, Katsushika Medical Center, 6-41-2 Aoto, Katsushika-ku, Tokyo, 125-8506 Japan; 6 Department of Neurosurgery, The Neurosurgery East Yokohama Hospital, 888 Hazawacho, Kanagawa-ku, Yokohama-City, Kanagawa 221-0863 Japan; 7 Department of Neurosurgery, Yokohama Shin-midori General Hospital, 1726-7 Tokaichibacho, Midori-ku, Yokohama-City, Kanagawa 226-0025 Japan; 8 Department of Fatigue Science, The Jikei University School of Medicine, 3-25-8 Nishi-Shimbashi, Minato-City, Tokyo 105-8461 Japan

**Keywords:** Intracranial aneurysm, Cerebral aneurysm, Stress, Stress marker, SITH-1, Biomarkers, Diseases, Medical research, Neurology, Neuroscience

## Abstract

The role of mental stress in intracranial aneurysm rupture remains unclear. Serum anti–SITH-1 antibody titers have been proposed as a potential biomarker of chronic mental stress. This study investigated the association between serum anti–SITH-1 antibody titers and intracranial aneurysm rupture. Between June 2021 and September 2023, patients with ruptured intracranial aneurysms (RIAs), patients with unruptured intracranial aneurysms (UIAs), and healthy controls were prospectively enrolled from five institutions. Baseline characteristics, aneurysm morphology, and lifestyle factors were recorded. Serum anti–SITH-1 antibody titers were quantified using a fluorescent antibody technique. Blood samples were obtained once in each participant: within 1 month after enrollment in the UIA and Control groups, and within 24 h of subarachnoid hemorrhage onset in the RIA group. The primary outcome was the association between serum anti–SITH-1 antibody titer and aneurysm rupture. Eighty-five participants were registered; after exclusions, 24 patients with RIAs were included for analysis, along with 26 patients with UIAs and 23 controls. Baseline characteristics were generally comparable, except for higher statin use in the UIA group (p = 0.012). Larger aneurysm size (p < 0.001), the presence of aneurysmal blebs (p < 0.001), and distinct aneurysm locations (p = 0.001) were more frequent in the RIA group. Anti–SITH-1 antibody titers differed significantly among groups (p = 0.008), being highest in the UIA group, followed by the RIA and Control groups. In the UIA group, antibody titers showed a significant positive correlation with time from diagnosis to study enrollment (p = 0.028). Anti–SITH-1 antibody titers, used as a marker of mental stress, were significantly higher in patients with UIAs than in those with RIAs or in healthy controls. These findings suggest that chronic mental stress is unlikely to play a critical role in aneurysm rupture but may be more prominent in individuals diagnosed with UIAs.

**Supplementary Information:**

The online version contains supplementary material available at 10.1038/s41598-026-42027-8.

## Introduction

Advances in imaging technologies have increased the likelihood of diagnosing unruptured intracranial aneurysms (UIAs) before rupture^1^^2^. Prospective epidemiological studies have reported an annual rupture rate of approximately 1%, with identified risk factors including age, aneurysm location, aneurysm size, presence of a daughter sac, geographical region, history of subarachnoid hemorrhage (SAH), hypertension, multiple aneurysms, and smoking^3–10^. In clinical practice, patients with a high epidemiological risk of rupture undergo surgical interventions such as craniotomy with clipping or endovascular coil embolization to prevent rupture, whereas those at low risk are managed conservatively with follow-up. However, some aneurysms classified as having a low risk of rupture may still rupture during follow-up, despite prophylactic management of modifiable risk factors such as hypertension and smoking. This suggests that the current risk assessments for UIA rupture may not be fully predictive.

Several studies have suggested a correlation between mental stress and stroke^11–16^. Among these, the Japan Collaborative Cohort Study for Evaluation of Cancer Risk (JACC Study), a large prospective epidemiological study conducted in Japan in the 1990 s, qualitatively demonstrated an association between mental stress and death due to SAH in women^17^^18^. However, those studies have relied on subjective assessments of mental stress, such as questionnaires, and no objective indicators have been available. The relationship between mental stress and intracranial aneurysm rupture thus remains unclear.

The small protein encoded by the intermediate-stage transcript of human herpesvirus 6 (SITH-1) has been identified as a latent infection protein of human herpesvirus (HHV)−6B. HHV-6B is reactivated in the olfactory bulb in response to mental stress or fatigue, leading to the production of SITH-1 protein. Since SITH-1 and its complexes induce apoptosis in neural stem cells and hippocampal neurons, elevated SITH-1 levels have been reported in patients with depression, but are rarely detected in healthy individuals^19^. The titer of anti-SITH-1 antibodies may thus offer an objective surrogate marker for mental stress. This study aimed to test the hypothesis that mental stress is associated with an increased risk of intracranial aneurysm rupture by quantitatively analyzing serum levels of anti-SITH-1 antibody.

## Methods

### Study design

Between June 2021 and September 2023, patients diagnosed with ruptured intracranial aneurysms (RIAs), patients diagnosed with unruptured intracranial aneurysms (UIAs), and healthy controls without intracranial aneurysm (confirmed through medical check-ups) were prospectively enrolled from five institutions (The Jikei University Hospital, Kashiwa Hospital, Katsushika Medical Center, The Neurosurgery East Yokohama Hospital, and Yokohama Shin-midori General Hospital). Written informed consent was obtained from all participants. When a patient’s condition precluded consent, consent was obtained from an appropriate surrogate.

Data collection was conducted by participating investigators in accordance with a predefined study protocol. This cohort study adhered to the STROBE guidelines for observational research. Ethical approval was obtained from the review board of each participating institution, with the following approval numbers: 32–476 and 33–365 for The Jikei University (covering three affiliated hospitals), 2021-005 for The Neurosurgery East Yokohama Hospital, and 2021.10-1.10.10 for Yokohama Shin-Midori General Hospital.

### Patients

This study included three groups: patients with RIAs (R group), patients with UIAs (U group), and healthy subjects (Control group). Patients in the R group met the following criteria: age between 20 and 80 years; confirmed diagnosis of an intracranial aneurysm based on cerebral vascular imaging (magnetic resonance angiography [MRA], computed tomography angiography [CTA], or digital subtraction angiography [DSA]), and presentation within 24 h of the estimated time of onset. Patients in the U group met the same age and diagnostic criteria as the R group, but all aneurysms were unruptured and had a maximum diameter < 5 mm. Patients in the Control group were also between 20 and 80 years old and had no evidence of intracranial aneurysms on cerebral vascular imaging.

Patients were excluded if they had a history of depression, genetic risk factors for intracranial aneurysm (including polycystic kidney disease, Ehlers–Danlos syndrome, Marfan syndrome, fibromuscular dysplasia, or moyamoya syndrome or disease)^20^, a family history of intracranial aneurysms (defined as two first-degree relatives with SAH or UIA)^21^, or a history of SAH.

We restricted the U group to aneurysms < 5 mm to reduce confounding by aneurysm size, which has been consistently identified as the strongest independent determinant of rupture risk in large prospective cohorts,⁶ and to more specifically explore the association between anti-SITH-1 antibody titers and the psychological burden of living with an unruptured aneurysm under surveillance. Because this study was hypothesis-driven rather than designed to achieve strict size-matched group comparisons, we prioritized reduction of biological confounding over numerical balance between groups.

### Data collection

Baseline characteristics were recorded for all patients, including age, sex, height, and weight, as well as lifestyle history. Lifestyle factors included smoking status (current, past, or never), alcohol consumption, and the presence of predisposing diseases such as hypertension, diabetes mellitus, dyslipidemia, congestive heart disease, and chronic renal failure. In addition, medication use was documented, including use of non-steroidal anti-inflammatory drugs, statins, steroids, anticoagulants, and antiplatelet agents.

Aneurysm characteristics were assessed based on images obtained from MRA, CTA, or DSA, and included size (neck diameter and height), location, and the presence of a bleb. The aspect ratio was calculated by dividing the aneurysm height by the neck diameter.

Blood samples were collected once in each participant. In the U and Control groups, samples were obtained within one month after enrollment. In the R group, samples were obtained within 24 h after SAH onset to minimize the influence of secondary systemic inflammatory responses, medical interventions, and time-dependent neuroendocrine changes associated with acute SAH. Because this study was designed to use a single time-point measurement across all groups, serial measurements were not performed. In addition to measuring anti-SITH-1 antibody titers, routine blood examinations were performed, including low-density lipoprotein cholesterol, high-density lipoprotein cholesterol, triglycerides, C-reactive protein, complete blood count, hemoglobin, and platelet count.

### Measurement of anti-SITH-1 antibody titer

The anti-SITH-1 antibody titer was quantified using a fluorescent antibody technique. As the antigen, a fusion protein of SITH-1 and the host protein calcium-modulating ligand (CAML) (N-SITH-CAML-C), previously described by Kobayashi et al.^19^, was purified.

A COS-7 cell line derived from the African green monkey was cultured, and pCMV-N-SITH-CAML-C was transfected using the Lipofectamine LTX Reagent with Plus Reagent (Thermo Fisher Scientific, Waltham, MA) according to the instructions from the manufacturer. Two days after transfection, SITH-1-CAML-expressing COS-7 cells were fixed with an acetone-methanol solution. These fixed cells were then incubated at 37 °C for 1 h with a mixture of a 10,000-fold dilution of anti-CAML antibody (#ab67714; Abcam, Cambridge, UK) and an 80-fold dilution of patient serum, prepared in diluent containing 0.05% Tween 20, 2% bovine serum albumin, and 0.08% sodium azide in phosphate-buffered saline (PBS). After washing with 0.2% Tween 20/PBS solution, cells were incubated with goat anti-human immunoglobulin (Ig)G (H + L) cross-adsorbed secondary antibody (Alexa Fluor 488, #A-11013; Thermo Fisher Scientific) and goat anti-mouse IgG (H + L) cross-adsorbed secondary antibody (Alexa Fluor 594, #A-11005; Thermo Fisher Scientific) at 37 °C for 30 min. Following incubation, cells were washed again with 0.2% Tween 20/PBS solution, embedded with ProLong Diamond Antifade Mountant with DAPI (#P36962; Thermo Fisher Scientific), and analyzed for fluorescence intensity using ArrayScan XT (Thermo Fisher Scientific).

The average fluorescence intensity of Alexa Fluor 488 was calculated as a measure of anti-SITH-1 antibody binding, while the average fluorescence intensity of Alexa Fluor 594 represented antigen expression based on anti-CAML antibody binding. To standardize anti-SITH-1 antibody binding, the fluorescence intensity of Alexa Fluor 488 was normalized by the corresponding Alexa Fluor 594 fluorescence intensity.

### Outcome

The primary outcome was to evaluate the association between mental stress and the risk of intracranial aneurysm rupture, as measured by serum anti-SITH-1 antibody levels. Secondary outcomes included assessing the relationship between mental stress and UIAs and examining the correlation between serum anti-SITH-1 antibody levels and the interval from aneurysm detection to antibody measurement.

### Statistical analysis

Comparisons among the R, U, and Control groups were conducted using appropriate statistical methods based on the type of variable. Categorical variables are presented as frequencies and percentages and were analyzed using Fisher’s exact test. Continuous variables are expressed as medians with interquartile ranges (IQRs) and were compared using the Kruskal–Wallis test. When a significant difference was observed, pairwise post hoc comparisons were performed with Bonferroni correction. Additionally, in the U group, the correlation between time from initial diagnosis to study enrollment and anti-SITH-1 antibody titer was analyzed using Spearman’s rank correlation coefficient. All statistical analyses were performed using Stata/SE version 17 (StataCorp LLC, College Station, TX, USA). Values of *p* < 0.05 were considered statistically significant.

## Results

**Fig. 1:**
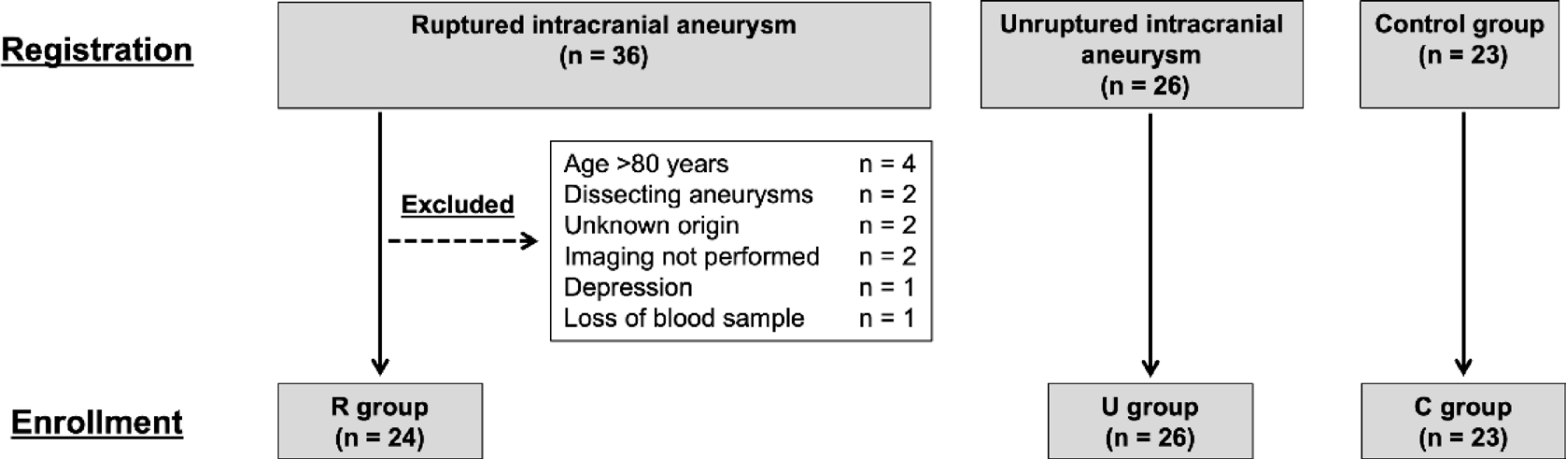
Patient registration, enrollment, and exclusion criteria. Eighty-five patients were registered for this study, comprising 36 in the R group, 26 in the U group, and 23 healthy subjects in the Control group. In the R group, several patients were excluded based on specific criteria: four were >80 years old, two had dissecting aneurysms, two had aneurysms of unknown origin, two did not undergo vascular imaging, one had a history of depression, and one patient was missing a blood sample. After these exclusions, 24 patients in the R group were enrolled for outcome assessment. R group: Patients with ruptured intracranial aneurysms. U group: Patients with unruptured intracranial aneurysms.

Eighty-five cases were registered for this study, comprising 36 patients in the R group, 26 in the U group, and 23 in the Control group. In the R group, patients were excluded based on the following criteria: age > 80 years (*n* = 4), presence of dissecting aneurysm (*n* = 2), unknown origin of SAH (*n* = 2), lack of vascular imaging (*n* = 2), history of depression (*n* = 1), and loss of blood sample (*n* = 1). Consequently, 24 patients in the R group were enrolled for outcome assessment Fig.1.

### Clinical background characteristics

Age, sex, body mass index, smoking status, and history of alcohol consumption, as well as pre-existing conditions including hypertension, diabetes mellitus, dyslipidemia, congestive heart disease, and chronic renal failure, did not differ significantly among the R, U, and Control groups (Table 1). Similarly, the use of medications such as non-steroidal anti-inflammatory drugs, steroids, anticoagulants, and antiplatelet agents showed no significant differences between groups. However, the proportion of patients taking statins was significantly higher in the U group compared with the R and Control groups (U: 27%, R: 0%, C: 8.7%; *p* = 0.012) (Table 1). In the R group, treatment modalities included microsurgical clipping (*n* = 14), endovascular coiling (*n* = 9), and conservative management (*n* = 1).

**Table 1:** Comparison of clinical background factors among ruptured patients, unruptured patients, and healthy controls.

	**R group (n=24)**	**U group (n=26)**	**Control (n=23)**	**p value**
Age, years	57.5 [48.8, 70.5]	55.0 [47.3, 58.8]	60.0 [55.0, 66.5]	0.086
Sex, female	15 (62.5)	14 (53.8)	8 (34.8)	0.15
Body mass index, kg/m^2^	21.5 [20.2, 23.1]	21.7 [20.4, 23.4]	23.1 [21.0, 24.4]	0.24
Smoking				0.16
Current	9 (40.9)	5 (19.2)	2 (9.1)	
Past	4 (18.2)	7 (26.9)	5 (22.7)	
Never	9 (40.9)	14 (53.8)	15 (68.2)	
Drinking	8 (36.4)	9 (34.6)	6 (26.1)	0.77
Pre-existing diseases				
Hypertension	10 (41.7)	6 (23.1)	6 (26.1)	0.36
Diabetes mellitus	0 (0)	1 (3.8)	3 (13.0)	0.11
Dyslipidemia	2 (8.3)	8 (30.8)	7 (30.4)	0.092
Congestive heart disease	0 (0)	0 (0)	0 (0)	NA
Chronic renal failure	1 (4.2)	0 (0)	0 (0)	0.64
Medication				
NSAID	1 (4.3)	0 (0)	0 (0)	0.65
Statin	0 (0)	7 (26.9)	2 (8.7)	0.012*
Steroid	1 (4.3)	0 (0)	0 (0)	0.65
Anticoagulant	1 (4.3)	0 (0)	1 (4.3)	0.53
Antiplatelet	0 (0)	2 (7.7)	0 (0)	0.33

NSAID, non-steroidal anti-inflammatory drugs. R group, patients with ruptured intracranial aneurism. U group, patients with unruptured intracranial aneurysm. *p < 0.05. Unless otherwise indicated, values represent the number of aneurysms (%) or median and interquartile range. Not all percentages total 100% due to rounding.

### Aneurysm characteristics and blood test results

The R group demonstrated significantly greater aneurysm height (5.0 mm) and aspect ratio (1.5) compared with the U group (2.3 mm and 0.9, respectively; both *p* < 0.001). The presence of aneurysmal blebs was also significantly more frequent in the R group (100%) than in the U group (8.3%; *p* < 0.001).

Aneurysm locations differed significantly between R and U groups (*p* = 0.001), as follows: posterior communicating artery (11 [46%] vs. 2 [7.7%]), internal carotid artery (2 [8.3%] vs. 13 [50%]), anterior communicating artery or anterior cerebral artery (6 [25%] vs. 3 [12%]), middle cerebral artery (3 [13%] vs. 7 [27%]), and vertebral or basilar artery (2 [8.3%] vs. 1 [3.8%]) (Table 2).

Blood test results showed no significant differences among the three groups in low-density lipoprotein cholesterol, high-density lipoprotein cholesterol, triglycerides, C-reactive protein, hemoglobin, or platelet count (Table 2). However, the white blood cell count was significantly higher in the R group compared with the U and Control groups (*p* < 0.001) (Table 2).

**Table 2:** Comparison of aneurysm characteristics and blood test results among ruptured patients, unruptured patients, and healthy controls.

	**R group (n=24)**	**U group (n=26)**	**Control (n=23)**	**p value**
Aneurysm characteristics				
Height, mm	5.0 [3.5, 6.3]	2.3 [1.7, 3.1]		< 0.001*
Neck, mm	3.0 [2.6, 3.9]	2.9 [2.3, 3.3]		0.25
Aspect ratio	1.5 [1.2, 1.7]	0.9 [0.6, 1.0]		< 0.001*
Bleb	23 (100)	2 (8.3)		< 0.001*
Location				0.001*
ICA	2 (8.3)	13 (50)		
ACoA/ACA	6 (25.0)	3 (12)		
MCA	3 (13)	7 (27)		
PCoA	11 (45.8)	2 (7.7)		
VA/BA	2 (8.3)	1 (3.8)		
Blood test				
LDL, mg/dL	122.0 [83.5, 154.0]	116.0 [104.3, 141.3]	116.5 [98.5, 130.3]	0.83
HDL, mg/dL	65.0 [58.3, 68.5]	68.0 [59.0, 75.0]	59.0 [54.3, 73.3]	0.52
TG, mg/dL	83.5 [65.0, 136.8]	107 [72.8, 180.0]	86.0 [63.3, 112.0]	0.52
CRP, mg/dL	0.05 [0.04, 0.13]	0.05 [0.04, 0.08]	0.04 [0.04, 0.08]	0.73
WBC, ´10^3^/μL	10.0 [6.6, 15.2]	5.0 [4.4, 5.6]	4.8 [4.4, 5.3]	< 0.001*
Hb, g/dL	14.4 [12.8, 14.9]	13.9 [13.1, 14.8]	14.3 [13.7, 15.1]	0.55
Plt, ´10^3^/μL	249 [188, 268]	251 [232, 276]	224 [214, 246]	0.16
Anti-SITH-1 antibody titer	1.32 [1.07, 2.14]	2.39 [1.68, 3.19]	1.39 [0.84, 1.98]	0.008*

ACA, Anterior cerebral artery; ACoA, Anterior communicating artery; Alb, Albumin;ALT, Alanine aminotransferase; AST, Aspartate aminotransferase; BA, Basilar artery; Cre, Creatinine; CRP, C-reactive protein; Hb, Hemoglobin; HbA1c, Hemoglobin A1c; HDL, High-density lipoprotein cholesterol; Ht, Hematocrit; ICA, Internal carotid artery; LDL, Low-density lipoprotein cholesterol; MCA, Middle cerebral artery; PCoA, Posterior communicating artery; Plt, Platelet count; SITH-1, Small protein encoded by the intermediate-stage transcript of HHV-6; T-Bil, Total bilirubin; TG, Triglycerides; TP, Total protein; UN, Urea nitrogen; VA, Vertebral artery; WBC, White blood cell count. *p < 0.05. Unless otherwise indicated, values represent the number of aneurysms (%) or median and interquartile range. Not all percentages total 100% due to rounding.

### Anti-SITH-1 antibody titer

**Fig. 2:**
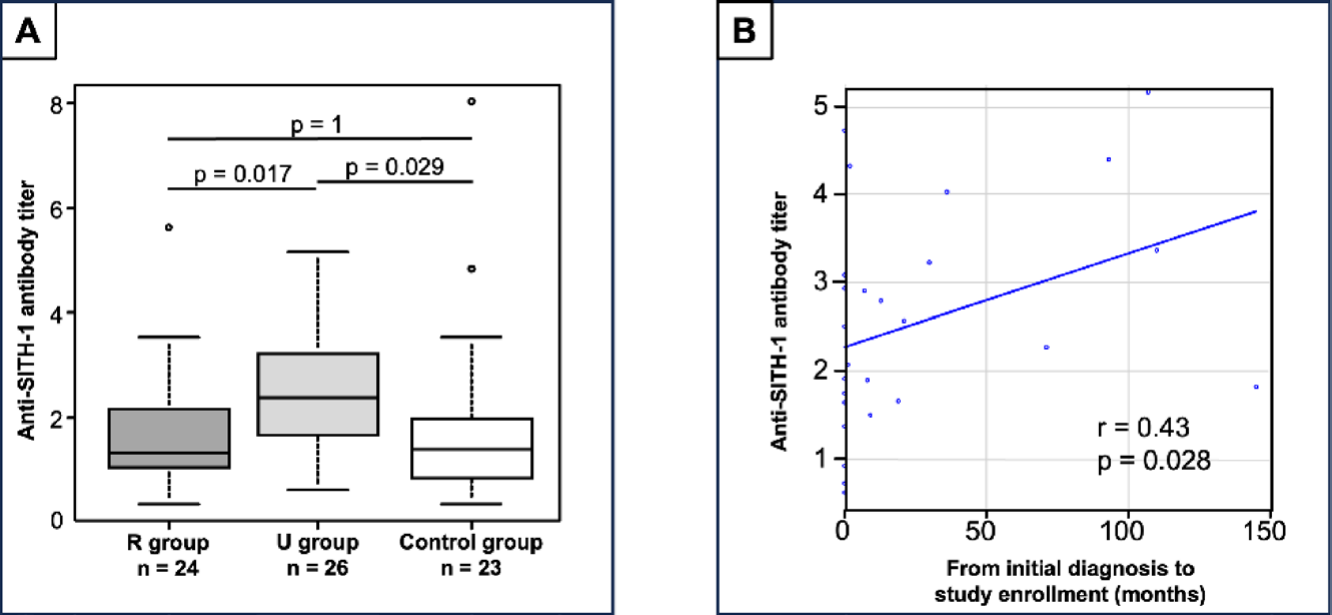
(**A**) Comparison of serum anti–SITH-1 antibody titers among three groups: patients with ruptured intracranial aneurysms presenting with subarachnoid hemorrhage (RIA group; blood sampled within 24 h of SAH onset), patients with unruptured intracranial aneurysms (UIA group), and healthy controls (Control group). (**B**) Correlation between serum anti–SITH-1 antibody titer and the interval from initial UIA diagnosis to study enrollment in the UIA group.

Median anti-SITH-1 antibody titers were significantly higher in the U group (2.39, IQR 1.68–3.19) compared with the R and Control groups (R group: 1.32, IQR 1.07–2.14; Control group: 1.39, IQR 0.84–1.98; combined *p* = 0.008) (Table 2). Post hoc pairwise comparisons showed no significant difference between R and Control groups (adjusted *p* = 1), but significant differences were identified between the U and R groups (adjusted *p* = 0.017) and between the U and Control groups (adjusted *p* = 0.029) (Fig. 2A). In the U group, anti-SITH-1 antibody titers showed a significant positive correlation with time from initial diagnosis to study enrollment (correlation coefficient: 0.43, *p* = 0.028) (Fig. 2B).

## Discussion

This study demonstrated that anti–SITH-1 antibody titers, used as a marker of mental stress, were not significantly associated with aneurysm rupture but were elevated in patients with UIAs. Furthermore, among patients with UIAs, anti–SITH-1 antibody titers showed a positive correlation with the interval from initial diagnosis to study enrollment. Collectively, these findings suggest that mental stress is unlikely to play a direct role in aneurysm rupture but may be more pronounced in individuals diagnosed with UIAs.

### SITH-1 as a potential biomarker of chronic mental stress

Kobayashi et al. recently identified SITH-1 as a latent HHV-6B protein specifically expressed in astrocytes. HHV-6B may proliferate and induce SITH-1 expression in response to stress or fatigue stimuli. In a mouse model, SITH-1 expression in olfactory astrocytes led to olfactory bulb apoptosis, hyperactivation of the hypothalamic–pituitary–adrenal (HPA) axis, and depressive symptoms. Notably, anti-SITH-1 antibody levels were significantly higher in patients with depression than in healthy controls (*p* = 1.78 × 10⁻¹⁵), with positivity rates of 79.8% and 24.4%, respectively (odds ratio, 12.2)^19^.

Although anti–SITH-1 antibody has primarily been reported in the context of depression, it was not applied in the present study as a diagnostic marker for any psychiatric disorder. Rather, it was utilized as an exploratory, biologically anchored surrogate marker that may reflect chronic stress- or fatigue-related physiological states. This rationale is based on prior experimental and clinical evidence linking stress- or fatigue-related stimuli to HHV-6B reactivation, subsequent SITH-1 expression, and downstream activation of the HPA axis. Therefore, anti–SITH-1 antibody levels in this study should be interpreted as a laboratory-based proxy of stress-related biological responses, rather than as an indicator specific to anxiety or adjustment disorders. Accordingly, its use should be regarded as exploratory rather than definitive. Collectively, these findings suggest that anti–SITH-1 antibody titers may potentially represent a candidate objective biomarker for quantifying chronic mental stress. Indeed, the previous study linking SITH-1 to depression demonstrated this association primarily through physiological and clinical correlates of depressive states, thereby lending biological plausibility to the interpretation of anti–SITH-1 antibodies as indicators of chronic stress–related biological responses^19^.

### Stress and cerebrovascular disease

Several studies have reported an association between psychological stress and stroke. The INTERSTROKE study, a multinational case–control study conducted across 22 countries, identified depression and psychosocial stress as significant risk factors for stroke^12^. Regarding the relationship between SAH and stress, the Japan Collaborative Cohort (JACC) study demonstrated that high mental stress constituted a prominent risk factor in women, with a population attributable risk of 13.1%^18^.

The proposed mechanisms linking stress to stroke involve dysregulation of the HPA axis, a pathway closely associated with stress and depression. Such dysregulation may result in elevated circulating catecholamines, impaired insulin sensitivity, endothelial dysfunction, and enhanced platelet activation^22–28^. Collectively, these pathophysiological changes may contribute to the development and progression of cerebrovascular disease, thereby increasing the risk of stroke across multiple subtypes^16^. However, in most previous studies, stress levels were assessed subjectively using self-reported questionnaires, and objective biomarkers for stress have not been established.

In the present study, titers of anti–SITH-1 antibody, used as a potential surrogate marker of chronic mental stress, were significantly higher in patients with UIAs than in those with RIAs or in healthy controls. In contrast, no significant difference was observed between patients with RIAs and healthy controls. These findings suggest that chronic mental stress may be associated with the presence of unruptured aneurysms, whereas it is unlikely to play a major role in aneurysm rupture.

### Unruptured intracranial aneurysm and mental stress

In the present study, patients with UIAs exhibited higher anti–SITH-1 antibody titers than those with RIAs. A meta-analysis reported prevalence rates of anxiety and depression of 28% and 21%, respectively, among patients with UIAs^29^. In addition, two prospective studies using the State–Trait Anxiety Inventory demonstrated that state anxiety—defined as transient anxiety elicited by specific situations—significantly decreased after UIA surgery, whereas trait anxiety, reflecting a stable personality characteristic, remained unchanged^30,31^.

Patients with untreated UIAs may experience heightened anxiety and mental stress related to concerns about aneurysm rupture and treatment-associated risks. Furthermore, routine surveillance and follow-up for UIAs may impose additional psychosocial burdens^32^. These factors may collectively contribute to the elevated anti–SITH-1 antibody titers observed in patients with UIAs. Notably, a positive correlation was identified between antibody titers and the interval from initial UIA diagnosis to study enrollment, further supporting a potential link between UIAs and chronic mental stress. This temporal relationship suggests a cumulative effect, whereby prolonged psychological stress may intensify over time in patients aware of their aneurysm, potentially leading to gradual increases in anti–SITH-1 antibody levels. Such a pattern strengthens the plausibility of SITH-1 as a dynamic biomarker reflecting the burden of persistent stress in this population.

### Statin use as a potential confounder

In the present study, statins were more frequently prescribed to patients with unruptured intracranial aneurysms (UIAs) than to those with ruptured intracranial aneurysms (RIAs). This imbalance likely reflects preventive medical management in clinically stable patients with UIAs; therefore, residual confounding cannot be excluded in this observational study.

A multicenter case–control study conducted in Japan reported an inverse association between statin use and intracranial aneurysm rupture (adjusted odds ratio [OR], 0.30; 95% confidence interval [CI], 0.14–0.66)^33^. Similarly, a large case–control study using propensity score–weighted multivariable analysis demonstrated a significant inverse association between lipid-lowering agent use and the risk of subarachnoid hemorrhage (OR, 0.41; 95% CI, 0.23–0.73)^34^. More recent studies have further corroborated the potential protective effect of statins in reducing the risk of aneurysm rupture^35^.

Although the present study was not designed to evaluate the effect of statins on rupture risk and adjustment for statin use was not feasible because of the limited sample size, these prior findings underscore statin use as a potentially important confounder that warrants careful consideration in larger, adequately powered studies. While statins have pleiotropic anti-inflammatory effects, their influence on anti–SITH-1 antibody levels remains unknown; therefore, the direction and magnitude of any potential bias cannot be determined in the present study.

### Research strengths and limitations

Key strengths of this study include its prospective design, multicenter collaboration, and systematic case stratification based on predefined criteria. Importantly, to our knowledge, this is the first study to evaluate anti–SITH-1 antibody titers in a non-psychiatric population.

Several limitations should also be acknowledged. First, the study was not conducted in a blinded manner. Second, the modest sample size limited statistical power and precluded multivariable analyses. Third, validated stress-related questionnaires were not employed. This was a deliberate design choice. In the acute SAH setting, the administration and interpretation of questionnaire-based assessments can be substantially confounded by impaired consciousness, neurological deficits, sedation, and acute emotional distress, making reliable evaluation difficult. Accordingly, we focused on an objective, laboratory-based surrogate marker that can be assessed even during the acute phase.

In addition, even in clinically stable patients with unruptured intracranial aneurysms, questionnaire scores are inherently state-dependent and may be influenced by transient emotional conditions, reporting bias, and cultural factors at the time of assessment, potentially limiting comparability across individuals and clinical contexts. In contrast, anti–SITH-1 antibody titers may represent a biologically anchored, integrated proxy of chronic stress- or fatigue-related physiological responses, allowing for more consistent single time-point comparisons across heterogeneous patient populations. We do not claim that this biomarker is superior to questionnaire-based assessments; rather, it should be viewed as a complementary objective indicator, particularly suitable when questionnaires cannot be reliably administered.

Fourth, because the UIA cohort was restricted to aneurysms < 5 mm, our findings may not be generalizable to patients with larger UIAs. Nevertheless, this restriction allowed a focused assessment of psychological stress in patients undergoing conservative surveillance, which represents a common clinical scenario.

Finally, because all participants were of Japanese ethnicity, the generalizability of these findings to other populations remains uncertain. Future studies involving larger, ethnically diverse cohorts are needed to confirm and extend these results. In addition, longitudinal investigations incorporating repeated anti–SITH-1 antibody measurements, together with aneurysm surveillance for growth and morphological changes and validated psychological assessments, are warranted to clarify temporal relationships and to determine whether this biomarker reflects the evolving psychological burden associated with living with UIAs. Such longitudinal designs would also help clarify whether anti–SITH-1 antibody levels remain stable over time or change in parallel with aneurysm growth or morphological progression, thereby further informing the relationship between this biomarker and aneurysm rupture.

## Conclusions

Anti–SITH-1 antibody titers, used as a marker of mental stress, were significantly higher in patients with UIAs than in those with RIAs or in healthy controls. These findings suggest that chronic mental stress is unlikely to play a critical role in aneurysm rupture but may be more prominent in individuals diagnosed with UIAs.

## Supplementary Information

Below is the link to the electronic supplementary material.

Supplementary Material 1

## Data Availability

The datasets used and/or analyzed during the current study are available from the corresponding author on reasonable request.

## Abbreviations

CTA: Computed tomography angiography
DSA: Digital subtraction angiography
MRA: Magnetic resonance angiography
RIA: Ruptured intracranial aneurysm
SAH: Subarachnoid hemorrhage
UIA: Unruptured intracranial aneurysm
